# Towards Understanding and Identification of Human Viral Co-Infections

**DOI:** 10.3390/v16050673

**Published:** 2024-04-25

**Authors:** Hui Wu, Hang-Yu Zhou, Heng Zheng, Aiping Wu

**Affiliations:** 1 School of Life Science and Technology, China Pharmaceutical University, Nanjing 211100, China; wuhui@stu.cpu.edu.cn; 2 Institute of Systems Medicine, Chinese Academy of Medical Sciences & Peking Union Medical College, Beijing 100005, China; 3 State Key Laboratory of Common Mechanism Research for Major Diseases, Suzhou Institute of Systems Medicine, Chinese Academy of Medical Sciences & Peking Union Medical College, Suzhou 215123, China

**Keywords:** human viral co-infections, viral interactions, host immune response, clinical symptoms, experimental and computational identification

## Abstract

Viral co-infections, in which a host is infected with multiple viruses simultaneously, are common in the human population. Human viral co-infections can lead to complex interactions between the viruses and the host immune system, affecting the clinical outcome and posing challenges for treatment. Understanding the types, mechanisms, impacts, and identification methods of human viral co-infections is crucial for the prevention and control of viral diseases. In this review, we first introduce the significance of studying human viral co-infections and summarize the current research progress and gaps in this field. We then classify human viral co-infections into four types based on the pathogenic properties and species of the viruses involved. Next, we discuss the molecular mechanisms of viral co-infections, focusing on virus–virus interactions, host immune responses, and clinical manifestations. We also summarize the experimental and computational methods for the identification of viral co-infections, emphasizing the latest advances in high-throughput sequencing and bioinformatics approaches. Finally, we highlight the challenges and future directions in human viral co-infection research, aiming to provide new insights and strategies for the prevention, control, diagnosis, and treatment of viral diseases. This review provides a comprehensive overview of the current knowledge and future perspectives on human viral co-infections and underscores the need for interdisciplinary collaboration to address this complex and important topic.

## 1. Introduction

Viruses are widespread in the biosphere and can infect all life forms, including humans, animals, plants, fungi, and bacteria [1,2,3]. They are obligate intracellular parasites that rely on host cells for replication and survival and can cause a wide range of diseases [4,5]. In recent years, several major viral outbreaks, such as Ebola, Zika, COVID-19, and monkeypox, have posed significant threats to global public health [6,7,8,9], highlighting the importance of understanding virus–host interactions and developing effective prevention and control strategies.

The human virome, which refers to the collection of all viruses that present in the human body, is large and complex [10,11]. It includes pathogenic viruses that usually cause various diseases directly, conditionally pathogenic viruses that normally establish asymptomatic or latent infections while exhibiting pathogenicity under certain circumstances, non-pathogenic viruses (i.e., bacteriophages), and endogenous retroviruses [12]. While most studies have focused on single virus infections, it is being increasingly recognized that viral co-infections, in which an individual is infected with multiple viruses simultaneously, are common in humans [13,14]. In addition to co-infections between multiple viruses, there is also the possibility of co-infections between viruses and other pathogens, such as fungi and bacteria [15,16,17,18]. Moreover, there is also the phenomenon of multiple infections, which is different from co-infection and deserves attention. This is a super-infection, which refers to a previously infected host being infected with different pathogens again [19,20,21]. In this review, we mainly focus on human viral co-infections; these viral co-infections can occur between different variants of the same virus, between different species of viruses, or between pathogenic and conditionally pathogenic viruses [22,23].

Viral co-infections can have profound impacts on viral evolution, host immunity, and disease outcomes [24]. Co-infecting viruses may interact directly or indirectly, leading to changes in viral replication, transmission, and pathogenesis. Moreover, viral co-infections can modulate host immune responses, causing enhanced or suppressed immunity, which may affect the clinical manifestations and severity of the disease [25,26,27,28,29,30,31,32,33,34,35,36,37,38,39,40]. Understanding the prevalence, types, mechanisms, and consequences of human viral co-infections is therefore crucial for the diagnosis, treatment, and prevention of viral diseases [41].

Despite the growing recognition of the importance of human viral co-infections, our understanding of this complex phenomenon remains limited. Current research mainly focuses on the impacts of specific viruses on the host, while the interactions between co-infecting viruses and their effects on host immunity are less well understood [21,24,27,28,29,30,31,32,33,34,35,36,37,38,39,40,42,43,44,45,46,47,48,49,50,51,52,53,54,55,56,57]. Moreover, the experimental detection and computational identification of viral co-infections can be challenging, especially for novel or emerging viruses [29,30,31,32,58,59,60,61,62,63,64,65,66,67,68,69,70,71,72]. Therefore, a comprehensive review of the current knowledge, methods, and gaps in human viral co-infection research is needed to guide future studies and inform clinical practice.

In this review, we aim to provide an overview of the types, mechanisms, impacts, and identification methods of human viral co-infections. We first introduce the diversity and complexity of the human virome and the significance of studying human viral co-infections. Then, we propose a classification system for human viral co-infections based on the pathogenic properties and species of the viruses involved. Next, we discuss the molecular mechanisms of viral co-infections, focusing on virus–virus interactions, host immune responses, and clinical manifestations, and provide examples from recent studies. We also summarize the experimental and computational methods of identifying viral co-infections, including multiplex polymerase chain reaction (PCR), immunoassays, metagenomics, and bioinformatics approaches. Finally, we highlight the challenges and future directions in human viral co-infection research and propose strategies for the integration of multi-omics data, the development of novel experimental models, and the fostering of interdisciplinary collaboration to advance our understanding and management of this important topic.

## 2. Types of Human Viral Co-Infection

**Figure 1:**
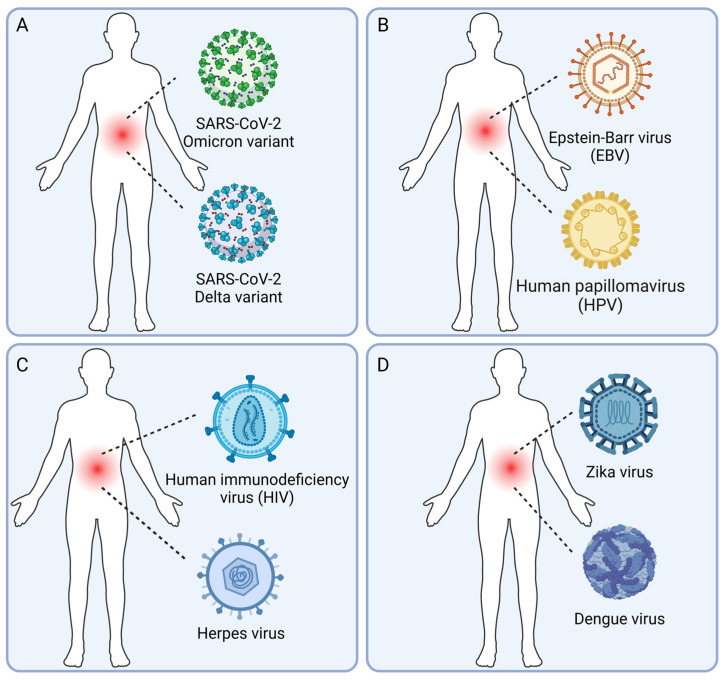
Types of human viral co-infection. (**A**) Co-infections with different variants of the same virus. (**B**) Co-infections with multiple conditionally pathogenic viruses. (**C**) Co-infections with pathogenic viruses and conditionally pathogenic viruses. (**D**). Co-infections with multiple pathogenic viruses. (Created with BioRender.com (accessed on 2 April 2024)).

The human virome is diverse and dynamic, and the pathogenic and conditionally pathogenic viruses in the human virome can establish acute, chronic, or latent infections [10,11]. Viral co-infections, in which multiple viruses infect a human being simultaneously, add another layer of complexity to this already intricate system. To better understand the scope and significance of human viral co-infections, we propose a classification system based on the pathogenic properties and species of the viruses involved (Figure 1).

### 2.1. Co-Infections with Different Variants of the Same Virus

This type of co-infection involves multiple variants or strains of the same virus species (Figure 1A), which may differ in their genetic sequences, antigenicity, virulence, or drug resistance [58,59,60,73,74,75,76,77,78,79,80,81,82,83,84,85,86,87,88,89,90,91,92,93,94]. Examples include co-infections with different subtypes of human immunodeficiency virus (HIV) [73,74,75,76,77], different lineages or reassortants of influenza virus [24,78,79,80,81,82,83,84], and different variants of severe acute respiratory syndrome coronavirus 2 (SARS-CoV-2) [58,59,60,85,86,87,88,89,90,91,92,93,94]. Co-infection with different viral variants can lead to competition or complementation between the variants and provide opportunities for recombination or reassortment, which may generate novel strains with altered biological properties [24,60,74,75,76,77,80,81,82,83,84,89,90,91,92,93,94].

The clinical implications of co-infection with different viral variants are complex and may vary depending on the specific virus and the host immune status. For example, co-infection with different HIV subtypes has been associated with faster disease progression and an increased viral load [74,75,76,77], while co-infection with different influenza virus subtypes may enhance viral shedding and transmission [80,81,82,83,84]. In the case of SARS-CoV-2, co-infection with different variants, such as Delta and Omicron, has been detected in several studies [58,59,86,87,88], but the clinical significance of these co-infections remains to be determined [59].

### 2.2. Co-Infections with Multiple Conditionally Pathogenic Viruses

Conditionally pathogenic viruses, also known as commensal or symbiotic viruses, are commonly found in healthy individuals and do not usually cause overt disease [22,23]. Examples include anelloviruses, circoviruses, and some herpesviruses [95,96,97], such as Epstein–Barr virus (EBV) and human cytomegalovirus (HCMV) [95,98,99,100]. These viruses may establish latent or persistent infections and coexist with the host and other viruses in a dynamic equilibrium [22,23,95,98,100,101].

Co-infections with multiple conditionally pathogenic viruses are likely common (Figure 1B), given the high prevalence of these viruses in the human population [10,11]. However, the interactions between these viruses and their impacts on host physiology and immunity are not well understood. Some studies have suggested that conditionally pathogenic viruses may modulate host immune responses and influence the outcomes of other infections or diseases [102,103,104]. For example, co-infection with EBV and human papillomavirus (HPV) has been associated with an increased risk of oral and nasopharyngeal cancers [103,104].

### 2.3. Co-Infections with Pathogenic Viruses and Conditionally Pathogenic Viruses

Pathogenic viruses, such as HIV, hepatitis B virus (HBV), and influenza virus, can cause severe and sometimes life-threatening diseases [21,29,30,31,42,43,44,45,73]. When a pathogenic virus co-infects with a conditionally pathogenic virus (Figure 1C), the interactions between the two viruses may have important clinical implications [25,26,27,28,105,106,107]. For example, HIV infection can reactivate latent herpesviruses, such as EBV, HCMV, and Kaposi’s sarcoma-associated herpesvirus (KSHV), leading to the development of opportunistic infections and cancers [25,26,27,28,108]. Similarly, co-infection with HBV and hepatitis D virus (HDV), a satellite virus that requires HBV for replication, can lead to more severe liver disease and accelerated progression to cirrhosis and liver cancer [109].

The mechanisms by which pathogenic viruses interact with conditionally pathogenic viruses are complex and may involve direct or indirect effects on viral replication, host cell metabolism, and immune responses [105,106,107]. Understanding these interactions is important for the management of human viral co-infections and the development of new therapeutic strategies [110].

### 2.4. Co-Infections with Multiple Pathogenic Viruses

Co-infections with multiple pathogenic viruses are of particular concern (Figure 1D), as they may lead to more severe clinical outcomes and pose challenges for diagnosis and treatment [21,29,30,31,32,33,34,35,36,37,38,39,40,42,43,44,45,46,47]. For example, patients co-infected with measles virus and influenza virus develop complications such as pneumonia or enteritis [46]. Zika virus (ZIKV), chikungunya virus (CHIKV), and dengue virus (DENV) infections result in similar clinical manifestations, but the mortality in patients co-infected with these viruses is higher [47].

The interactions between co-infecting pathogenic viruses can be complex and may involve competition for host resources, the modulation of immune responses, and the alteration of viral replication and pathogenesis [21,42,43,44,45]. For example, co-infection with HIV and HCV is associated with the faster progression of liver disease, a higher HCV viral load, and an increased risk of liver-related mortality [29,30,32,33]. Similarly, co-infection with RSV and other respiratory viruses can lead to more severe respiratory symptoms, prolonged hospitalization, and an increased risk of mortality, especially in young children and older adults [50,111,112,113,114].

The diagnosis and treatment of co-infections with multiple pathogenic viruses can be challenging, as the symptoms may overlap and the interactions between the viruses may affect the efficacy of antiviral drugs, and drug therapy may also affect co-infected viruses [21,34,115]. For example, in HBV/HCV co-infected patients, direct-acting antiviral agent (DAA) therapy against HCV infection sometimes causes HBV reactivation [116]. Therefore, a better understanding of the prevalence, types, and mechanisms of these co-infections is crucial for the development of effective prevention and control strategies [110,117].

## 3. Molecular Mechanisms and Clinical Impacts of Human Viral Co-Infection

**Figure 2:**
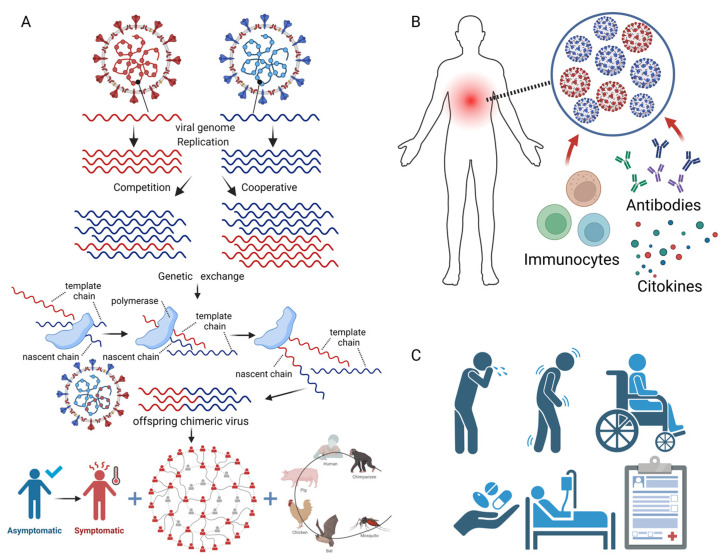
Molecular and clinical impacts of human viral co-infection. (**A**) Virus–virus interactions. Interactions between co-infected viruses include competition, cooperation, and gene exchange. Competition and synergy can manifest as the inhibition and enhancement of viral genome replication. Gene exchange, which involves recombination or reassortment, is one of the major ways in which viruses evolve. The mechanism of viral recombination is relatively complex, and a common and widely accepted approach is “copy choice” recombination. During the process of viral replication, the polymerase moves from one template chain to another while remaining bound to the nascent chain, resulting in offspring viruses with chimeric genomes. The offspring chimeric virus may have stronger biological characteristics, including stronger virulence, causing more severe clinical symptoms, a stronger transmission ability, the outbreak of pandemics in populations, and even cross-species transmission. (**B**) Host immune responses. (**C**) Clinical manifestations of disease. (Created with BioRender.com (accessed on 2 April 2024)).

Viral co-infections involve complex interactions between the co-infecting viruses, the host cells, and the immune system, which can have profound impacts on viral replication, pathogenesis, and disease outcomes (Figure 2 and Table 1). In this section, we discuss the molecular mechanisms of viral co-infections, focusing on virus–virus interactions, host immune responses, and clinical manifestations, and provide examples from recent studies.

### 3.1. Virus–Virus Interactions

Co-infecting viruses can interact directly or indirectly, leading to changes in viral replication, gene expression, and evolution [61,111,118,119,121,123,124,125] (Figure 2A). These interactions can be competitive, whereby one virus suppresses the replication or pathogenesis of the other [14], or cooperative, whereby one virus enhances the replication or pathogenesis of the other [61,121]. For example, co-infections with human rhinovirus and other respiratory viruses can lead to competitive interactions, with rhinovirus suppressing the replication of other respiratory viruses [111]. In contrast, co-infection with HIV and HCV can lead to cooperative interactions, with HIV enhancing the replication and pathogenesis of HCV [29,30].

The mechanisms of virus–virus interactions are complex and may involve direct physical interactions between viral proteins, competition for host cell receptors or replication machinery, or the modulation of host cell signaling pathways. For example, co-infection with human cytomegalovirus (HCMV) and herpes simplex virus-1 (HSV-1) can lead to the enhanced replication of both viruses, possibly through direct interactions between viral proteins or the modulation of the host cell metabolism [121]. Similarly, co-infection with human parainfluenza virus type 2 (hPIV2) and influenza A virus (IAV) can lead to enhanced IAV replication, possibly through hPIV2-induced changes in host cell fusion and membrane permeability [61].

Viral co-infections also provide opportunities for genetic exchange between the co-infecting viruses, through mechanisms such as recombination or reassortment [118,119,123,124,125] (Figure 2A). Recombination involves the exchange of genetic material between two closely related viruses, while reassortment involves the exchange of entire gene segments between two viruses with segmented genomes, such as influenza viruses [119,124]. These processes can generate novel viral strains with altered antigenicity, virulence, or host ranges and contribute to viral evolution and emergence [118,119,123,124,125]. For example, recombination between different subtypes of HIV can lead to the emergence of novel strains with increased fitness and drug resistance [118], while reassortment between human and avian influenza viruses can lead to the emergence of pandemic strains [119].

### 3.2. Host Immune Responses

Viral co-infections can have complex and sometimes opposing effects on host immune responses, leading to enhanced or suppressed immunity [112,120,122] (Figure 2B). The specific immune responses elicited by viral co-infections depend on the types of viruses involved, the timing and order of the infections, and the host immune status [41].

In some cases, co-infection with two viruses can lead to enhanced immune responses, possibly through cross-reactive T-cell or antibody responses [112,122]. For example, co-infection with influenza A virus and respiratory syncytial virus (RSV) can lead to the enhanced activation of natural killer (NK) cells and CD8+ T cells, which may provide cross-protection against subsequent infections [112,126]. Similarly, co-infection with SARS-CoV-2 and influenza virus can lead to the enhanced activation of neutrophils and the production of pro-inflammatory cytokines, which may contribute to the development of severe disease [122].

In other cases, viral co-infections can lead to suppressed or dysregulated immune responses, possibly through the induction of immunosuppressive cytokines or the exhaustion of antiviral T cells [25,26,27,28,120]. For example, co-infection with HIV and herpesviruses, such as EBV, HCMV, and KSHV, can lead to the reactivation of the herpesviruses and the development of opportunistic infections and cancers, due to the immunosuppressive effects of HIV [25,26,27,28]. Similarly, co-infection with HCV and other hepatitis viruses can lead to the dysregulation of the innate and adaptive immune responses in the liver, contributing to the development of chronic liver disease and cancer [120].

The long-term effects of viral co-infection on host immunity are not well understood and may depend on the specific viruses and host factors involved. Some studies have suggested that viral co-infections may lead to the development of chronic inflammation or autoimmunity, while others have suggested that they may lead to the induction of regulatory T cells or the establishment of viral persistence. Further research is needed to elucidate the complex interactions between viral co-infections and host immunity, and to develop new strategies for the prevention and treatment of co-infection-associated diseases [127].

### 3.3. Clinical Manifestations

The clinical manifestations of viral co-infections are highly variable and depend on the types of viruses involved, the timing and order of the infections, and the host immune status (Figure 2C). In some cases, viral co-infections can lead to more severe or prolonged symptoms compared to single infections, while, in other cases, they may have no apparent effect on the disease outcomes [25,26,27,28,46,47,48,49,50,51,52,53,54,55,56,57].

Studies have shown that co-infections with multiple respiratory viruses, such as influenza virus, RSV, and rhinovirus, can lead to more severe respiratory symptoms, prolonged hospitalization, and an increased risk of mortality, especially in young children and older adults [21,42,43,44,45,48,50,51]. Similarly, co-infections with HIV and conditionally pathogenic viruses, such as herpesviruses and human papillomavirus (HPV), can lead to the development of AIDS-defining illnesses and cancers [25,26,27,28].

In contrast, some studies have found no significant differences in disease severity between single and co-infections, or even reduced severity in co-infected patients [54,55,56,57]. For example, a study of children with bronchiolitis found no significant differences in clinical outcomes between those with single virus infections and those with multiple virus infections [55]. Another study of children with acute respiratory infections found that co-infections with multiple respiratory viruses were associated with the reduced severity of symptoms compared to a single respiratory virus infection [57].

The discrepancies in the clinical outcomes of human viral co-infections may reflect the complex interactions between the viruses, the host, and the environment, as well as the limitations of current diagnostic and classification methods. For example, the use of different diagnostic tests, such as PCR or serological assays, may affect the detection and interpretation of viral co-infections [29,30,31,32,61,62,63,64,65]. Similarly, the lack of standardized definitions and classification systems for viral co-infections may hinder the comparison and generalization of results across studies [128].

To better understand the clinical impacts of human viral co-infections, future studies should use standardized diagnostic methods and classification systems and consider the potential confounding effects of host and environmental factors. Moreover, the development of novel experimental models and computational tools for the study of viral co-infections may provide new insights into the mechanisms and outcomes of these complex co-infections [111].

## 4. Experimental and Computational Identification of Human Viral Co-Infections

The accurate and timely identification of human viral co-infections is crucial for the diagnosis, treatment, and prevention of the associated diseases. However, the detection and characterization of multiple viruses in a single sample can be challenging, due to the limitations of traditional diagnostic methods and the complexity of viral genomes and interactions [29,30,31,32,61,62,63,64,65]. In this section, we review the current experimental and computational methods for the identification of viral co-infections, and discuss their advantages, limitations, and future perspectives.

### 4.1. Experimental Methods for the Identification of Viral Co-Infections

**Figure 3:**
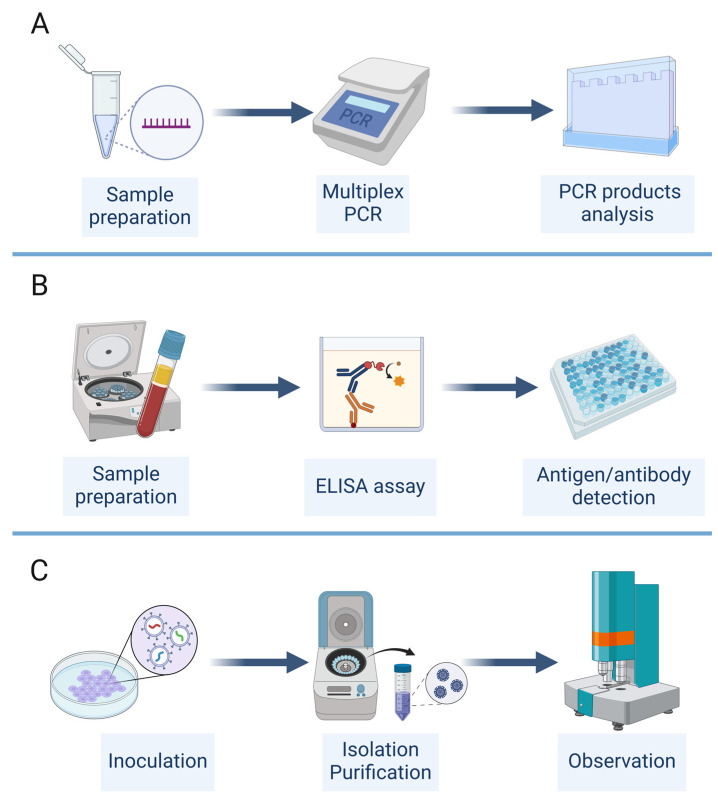
Experimental detection methods for viral co-infections. (**A**) PCR test. (**B**) Immunological assay. (**C)** Virus culture. (Created with BioRender.com (accessed on 17 April 2024)).

Several experimental methods have been used for the detection and characterization of viral co-infections, including multiplex PCR, immunological assays, and virus culture (Figure 3). Each of these methods has its own advantages and limitations, and the choice of method depends on the specific viruses and samples involved, as well as the available resources and expertise.

Multiplex PCR is a widely used method for the simultaneous detection of multiple viral targets in a single reaction [62,63,64] (Figure 3A). This method involves the use of multiple primer sets that are specific to different viral genes or regions, allowing the amplification and detection of multiple viruses in a single assay. Multiplex PCR can be performed using conventional or real-time PCR platforms and can detect a wide range of viruses, including RNA and DNA viruses [62,63,64]. One example is FilmArray, which can detect multiple viruses simultaneously, and even bacteria, fungi, and parasites, depending on the apparatus involved [129]. The advantages of multiplex PCR include its high sensitivity, specificity, and throughput, as well as its ability to detect multiple viruses in a single sample. However, the design and optimization of multiplex PCR assays can be challenging, due to the potential for primer–primer interactions and competition for reagents, which may affect the sensitivity and specificity of the assay.

Immunological assays, such as the enzyme-linked immunosorbent assay (ELISA), Western blot, and immunofluorescence assay (IFA), are also commonly used for the detection of viral co-infections [29,30,31,32,61,65] (Figure 3B). These assays involve the use of antibodies that are specific to viral antigens or proteins, allowing the detection and quantification of multiple viruses in a single sample [65]. The advantages of immunological assays include their high specificity, ease of use, and ability to detect both active and past infections. However, the sensitivity of these assays may be lower than that of PCR-based methods, and the interpretation of the results may be complicated by cross-reactivity between different viral antigens.

Virus culture is a traditional method for the isolation and characterization of viruses from clinical samples [14,61] (Figure 3C). This method involves the inoculation of a sample onto a permissive cell line, followed by the observation of cytopathic effects and the identification of the virus using specific antibodies or molecular methods. Virus culture can be used to detect multiple viruses in a single sample and can provide information on the infectivity and replication kinetics of the viruses [61]. However, virus culture is time-consuming, labor-intensive, requires specialized expertise and facilities, and must be carried out in corresponding biosafety-level laboratories [14]. Not all laboratories have the conditions for virus culture. Moreover, some viruses may not grow well in a cell culture or may require the use of specific cell lines or growth conditions [14].

### 4.2. Computational Methods for the Identification of Viral Co-Infections

**Figure 4:**
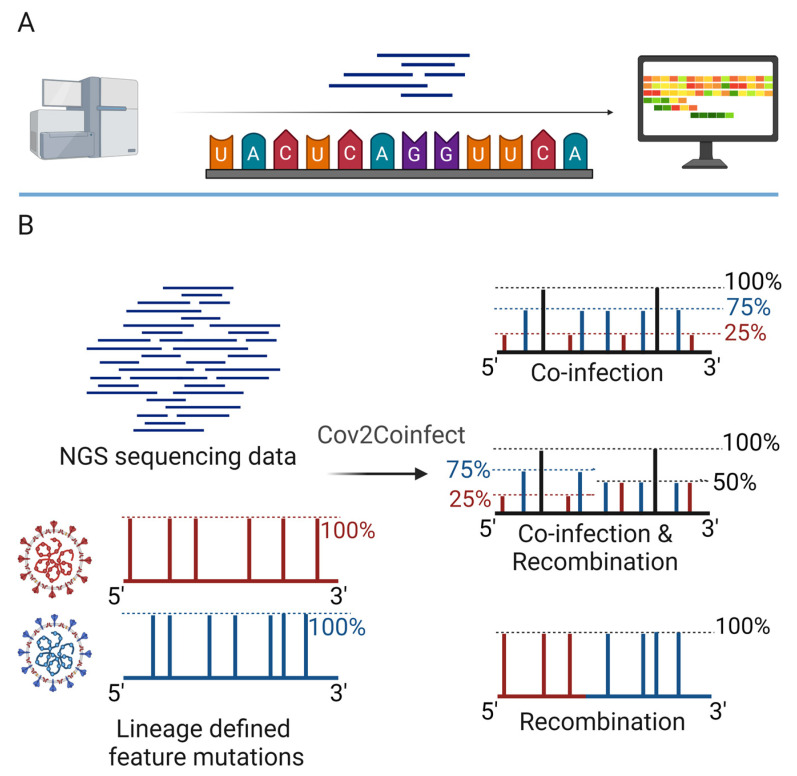
Computational identification methods for viral co-infections. (**A**) Genome sequencing. (**B**) Cov2Coinfect algorithm. The input data (both NGS sequencing data and lineage-defined feature mutation list) are sent for a hypergeometric distribution test to obtain candidate lineages. Then, the confirmed lineage composition is identified from the candidate lineages based on the genomic characteristics of the co-infection samples. When a patient is infected by one specific SARS-CoV-2 lineage, most of the feature mutations of the lineage are detected at the same frequency. When a patient is co-infected by two SARS-CoV-2 lineages, the feature mutations of the same lineage have similar frequencies; the sum of the average frequencies of the feature mutations unique to the two lineages is close to 100% and the frequency of the feature mutations shared by two lineages is close to 100%. When two co-infected lineages recombine in vivo, the feature mutations of the two co-infected lineages can be detected at a frequency of about 100% before and after the recombination breakpoint in the recombinant virus. Therefore, when two co-infected lineages recombine in a patient, changes in the frequency of the feature mutations of the two lineages before and after the recombination breakpoint can be observed (one increases, one decreases, and the sum remains at nearly 100%). (Created with BioRender.com (accessed on 2 April 2024)).

In recent years, the development of high-throughput sequencing technologies and bioinformatics tools has revolutionized the identification and characterization of viral co-infections [66,67,68,69,70,71,72] (Figure 4). These methods allow the unbiased detection and analysis of all viruses present in a sample, including novel and emerging viruses.

Metagenomics is a powerful approach for the identification of viral co-infections, as it involves the sequencing of all nucleic acids present in a sample (Figure 4A), without the need for prior knowledge of the viral genomes. This approach can detect both known and novel viruses, as well as their genetic variants and recombinants [70,71,72]. Metagenomics studies have revealed the high prevalence and diversity of viral co-infections in various human tissues and body fluids, including the respiratory tract, gut, and blood. However, the analysis of metagenomic data can be challenging, due to the large amount of data generated, the presence of host and bacterial sequences, and the need for specialized bioinformatics tools and databases.

Several bioinformatics tools and platforms have been developed for the analysis of viral metagenomic data, including GISAID [66,67,68], the Virus Variation Resource [69], and 2019nCoVR [71]. These platforms provide access to large databases of viral genomes and annotations, as well as tools for the assembly, alignment, and phylogenetic analysis of viral sequences [66,67,68,69,70,71,72]. These tools can be used to identify and characterize viral co-infections, as well as to track the evolution and transmission of viruses over time and space [69,70,71,72].

Computational methods have also been developed for the specific identification of viral co-infections and recombinants from sequencing data [58,59,60]. For example, the Cov2Coinfect algorithm uses a hypergeometric distribution test to identify the co-occurrence of lineage-defining feature mutations in SARS-CoV-2 genomes (Figure 4B), allowing the detection of co-infections with different viral variants [58]. Similarly, a method developed by Pipek et al. uses the unique defining mutations of viral variants and mutually exclusive defining mutations in variant combinations to identify co-infections and intra-host recombination in SARS-CoV-2 samples [59]. These methods provide a rapid and accurate way to detect viral co-infections and recombinants and can be applied to large datasets of viral genomes.

Despite the advances in experimental and computational methods for the identification of viral co-infections [62,63,64,65,66,67,68,69,70,71,72], several challenges and limitations remain. For example, the sensitivity and specificity of these methods may be affected by the quality and quantity of the viral nucleic acids in the sample, as well as by the presence of PCR inhibitors or contaminating sequences [62,63,64]. Moreover, the interpretation of the results may be complicated by the potential for cross-reactivity between different viral targets, or by the presence of defective or incomplete viral genomes [69,70,71,72].

## 5. Discussion and Future Perspectives

Human viral co-infections are a complex and understudied phenomenon that have important implications for viral evolution, host immunity, and disease outcomes. Despite the growing recognition of the prevalence and significance of viral co-infections, our understanding of their types, mechanisms, impacts, and identification methods remains limited. In this review, we have provided an overview of the current knowledge and gaps in the field of human viral co-infection and highlighted the need for further research and collaboration to address this important public health challenge.

We have proposed a classification system for human viral co-infections based on the pathogenic properties and species of the viruses involved, which includes co-infections with different variants of the same virus, co-infections with multiple conditionally pathogenic viruses, co-infections with pathogenic and conditionally pathogenic viruses, and co-infections with multiple pathogenic viruses. This classification system provides a framework for the study of the different types and consequences of viral co-infections, and for the development of targeted prevention and control strategies.

We have also discussed the molecular mechanisms of viral co-infections, including virus–virus interactions, host immune responses, and clinical manifestations. Virus–virus interactions can be competitive or cooperative and can involve direct physical interactions, competition for host resources, or the modulation of host cell signaling pathways. These interactions can affect viral replication, evolution, and pathogenesis and provide opportunities for the emergence of novel viral strains [118,119,123,124,125]. Host immune responses to viral co-infections can be enhanced or suppressed, depending on the types of viruses involved, the timing and order of the infections, and the host immune status [25,26,27,28,112,120,122]. These responses can affect the susceptibility to and severity of viral diseases, as well as the development of chronic inflammation or autoimmunity. The clinical manifestations of viral co-infections are highly variable, ranging from asymptomatic infections to severe and life-threatening diseases [25,26,27,28,46,47,48,49,50,51,52,53,54,55,56,57]. The accurate diagnosis and management of viral co-infections require a better understanding of their types, mechanisms, and impacts, as well as the development of standardized diagnostic and classification methods.

It is often difficult to identify co-infections as viruses are detectable from different types of samples and with different methods. We have reviewed the current experimental and computational methods for the identification of viral co-infections, including multiplex PCR, immunological assays, virus culture, metagenomics, and bioinformatics tools [62,63,64,65,66,67,68,69,70,71,72]. These methods have different advantages and limitations; for example, most multiplex PCRs are qualitative, making it difficult to detect the most relevant pathogen in a co-infection. In fact, being very sensitive assays, they can also detect “traces” of a virus that caused a now resolved infection, which can interfere with the detection of co-infections. The choice of method depends on the specific viruses and samples involved, as well as the available resources and expertise [62,63,64,65]. The integration of multiple approaches, such as the combination of PCR and sequencing methods, may help to improve the accuracy and reliability of viral co-infection identification. Furthermore, the finding of a positive result often interrupts the detection process to search for other co-infected viruses as the symptoms may be attributed to the first detected virus. Moreover, the development of novel experimental models, such as organoids and animal models, may provide new insights into the mechanisms and consequences of viral co-infections.

Despite the advances in viral co-infection research, several challenges and opportunities remain. First, the complex interactions between co-infecting viruses, host cells, and immune responses are still poorly understood, and they require further investigation using systems biology and multi-omics approaches. Second, the long-term impacts of viral co-infections on host health and disease risk are largely unknown, and they require longitudinal studies and surveillance programs. Third, the development of effective vaccines and therapies for viral co-infections is hindered by the lack of suitable animal models and the potential for drug–drug interactions. Fourth, the public health and socioeconomic burden of viral co-infections, especially in low- and middle-income countries, is underestimated and requires urgent attention and action.

To address these challenges and opportunities, we propose several future research directions and strategies. First, the integration of multi-omics data, such as genomics, transcriptomics, proteomics, and metabolomics, may provide a comprehensive view of the molecular mechanisms and consequences of viral co-infections. Second, the development of novel experimental models, such as organoids, humanized mice, and non-human primates, may enable the study of viral co-infections in a more physiologically relevant context. Third, the establishment of international collaborations and data sharing platforms, such as GISAID and the Global Virome Project, may facilitate the rapid detection, characterization, and response to viral co-infections, especially during outbreaks and pandemics. Fourth, the engagement of multidisciplinary teams, including virologists, immunologists, clinicians, epidemiologists, and bioinformaticians, may accelerate the translation of basic research findings into clinical and public health applications.

In conclusion, human viral co-infections represent a significant challenge and opportunity for biomedical research and public health. By integrating cutting-edge experimental and computational methods and fostering collaboration and data sharing across disciplines and borders, we can improve our understanding and management of these complex infections and develop more effective strategies for their prevention, diagnosis, and treatment. This review provides a comprehensive and up-to-date overview of the field of human viral co-infections and highlights the need for further research and investment in this important area.

## Figures and Tables

**Table 1:** Summary of well-known virus co-infection combinations and their impacts.

Viruses	Impacts	Reference
different subtypes of HIV	faster disease progression and increased viral load, recombination	[74,75,76,77,118]
different subtypes of influenza virus	enhanced viral shedding and transmission, reassortment	[80,81,82,83,84,119]
different variants of SARS-CoV-2	clinical significance remains to be determined, recombination	[58,59,86,87,88]
EBV/HPV	increased risk of oral and nasopharyngeal cancers	[103,104]
HIV/herpesviruses	development of opportunistic infections and cancers	[25,26,27,28,108]
HBV/HDV	more severe liver disease and accelerated progression to cirrhosis and liver cancer	[109]
measles virus/influenza virus	development of complications such as pneumonia or enteritis	[46]
ZIKV/CHIKV/DENV	higher mortality	[47]
HIV/HCV	HIV enhances replication and pathogenesis of HCV, faster progression of liver disease, higher HCV viral load, and increased risk of liver-related mortality	[29,30,32,33]
RSV/other respiratory viruses	more severe respiratory symptoms, prolonged hospitalization, and increased risk of mortality	[50,111,112,113,114]
HBV/HCV	DAA therapy against HCV infection may cause HBV reactivation	[116]
HCV/other hepatitis viruses	dysregulation of innate and adaptive immune responses in liver, contributing to development of chronic liver disease and cancer	[120]
rhinovirus/other respiratory viruses	rhinovirus suppressing replication of other respiratory viruses	[111]
HCMV/HSV-1	enhanced replication of both viruses	[121]
hPIV2/IAV	enhanced IAV replication	[61]
SARS-CoV-2/influenza virus	enhanced activation of neutrophils and production of pro-inflammatory cytokines, severe disease	[122]

## Data Availability

Not applicable.
